# Carbapenem resistance and antibacterial potential of the Libyan endemic plant Arbutus pavarii against metallo-β-lactamase-producing Gram-negative bacteria

**DOI:** 10.1099/acmi.0.001116.v3

**Published:** 2025-12-16

**Authors:** Mohanned Mohamed Alwashaish, Retaj Bashir Erhooma, Zainab Ahmed Taher, Dania Nuri Elhessan

**Affiliations:** 1 Microbiology Department, Faculty of Pharmacy, Misurata University, Misurata, Libya

**Keywords:** antimicrobial resistance, *Arbutus pavarii*, carbapenem resistance, Libya, metallo-β-lactamase (MBL)

## Abstract

**Background.** Carbapenem-resistant Gram-negative bacteria (CR-GNB), particularly metallo-β-lactamase (MBL) producers, are WHO critical-priority pathogens. In Libya, laboratory-based data are scarce, and no study has assessed endemic medicinal plants as adjunctive options.

**Objectives.** To generate baseline data on carbapenem resistance and MBL production among clinical Gram-negative pathogens in Misurata, Libya, and to preliminarily evaluate the antibacterial activity and phytochemical composition of *Arbutus pavarii* extracts.

**Methods.** We conducted a cross-sectional study of 244 non-duplicate clinical isolates. Carbapenem susceptibility was determined by Kirby–Bauer disc diffusion; MBL production was confirmed by double-disc synergy and combined-disc tests. Ethanolic and aqueous extracts from *A. pavarii* leaves, stems and fruits were tested against resistant isolates by disc diffusion. Phytochemicals were profiled by HPLC.

**Results.** The predominant carbapenem-resistant species were *Acinetobacter baumannii* (29.5%), *Klebsiella pneumoniae* (26.2%) and *Pseudomonas aeruginosa* (19.7%). Resistance to both imipenem and meropenem exceeded 60% across these isolates, and MBL activity was detected in 54.5% of carbapenem-resistant *K. pneumoniae*. Among plant extracts, the aqueous leaf extract showed the highest antibacterial activity against MBL-producing isolates (mean inhibition zone 9.46±7.61 mm at 100%), slightly exceeding the corresponding ethanolic extract (9.31±7.30 mm). Both extracts demonstrated concentration-dependent effects (*P*<0.05; ANOVA/Kruskal–Wallis). HPLC analysis identified catechin and quercetin as major components, which may underlie the observed activity.

**Conclusions.** This first laboratory-based report from Libya documents high rates of CR-GNB and MBL production and introduces *A. pavarii* as a promising endemic plant with adjunctive antibacterial potential. Findings support enhanced AMR surveillance and the exploration of resource-sensitive alternatives in African healthcare settings.

## Graphical Abstract

A. pavarii extracts against carbapenem-resistant Acinetobacter, Klebsiella, and Pseudomonas with >60% resistance. Ethanolic extracts containing catechin and quercetin show higher antibacterial activity than aqueous extracts in Libya.

## Data Summary

All data supporting the findings of this study are available as supplementary material (Table S1) submitted with the manuscript.

## Introduction

Antimicrobial resistance (AMR) is one of the most critical threats to global health, currently responsible for an estimated 1.27 million deaths annually and projected to cause up to 10 million deaths per year by 2050 if unmitigated [1]. Among resistant organisms, Gram-negative bacteria pose the greatest concern due to their intrinsic defenses and high capacity for horizontal gene transfer [2,3]. Species such as *Klebsiella pneumoniae*, *Pseudomonas aeruginosa* and *Acinetobacter baumannii* are major causes of healthcare-associated infections, particularly in intensive care settings, and exhibit alarming rates of multidrug resistance [4,5].

Carbapenems have long been considered last-resort agents; however, the global rise of carbapenem-resistant Gram-negative bacteria (CR-GNB) severely compromises their utility [6]. Metallo-β-lactamases (MBLs), including NDM, VIM and IMP families, hydrolyze nearly all β-lactams and are not inhibited by available β-lactamase inhibitors, making infections exceptionally difficult to treat [7,8]. The WHO has therefore classified CR-GNB, especially MBL-producers, as critical-priority pathogens [9].

In Libya, carbapenem resistance among hospital isolates is increasing, yet most studies remain descriptive and lack systematic evaluation of resistance mechanisms [10,11]. At the same time, there is an urgent need to explore locally available, cost-effective therapeutic alternatives. *Arbutus pavarii* Pamp., an evergreen shrub endemic to Jebel Al-Akhdar, Libya, is traditionally used to manage gastrointestinal and urinary infections [12]. Phytochemical studies report high polyphenolic content with antioxidant and antimicrobial properties [13,14], but its efficacy against clinical MBL-producing CR-GNB remains unexplored.

This study therefore addresses two critical gaps: (i) it characterizes carbapenem resistance and MBL production among Libyan clinical isolates, and (ii) it evaluates, for the first time, the antibacterial activity of *A. pavarii* extracts against MBL-producing CR-GNB.

## Methods

### Study design and isolates

This cross-sectional study was conducted at Misurata Medical Center between October 2024 and January 2025. A total of 244 non-duplicate Gram-negative isolates were collected from patients with suspected nosocomial infections as part of routine diagnostic care. No identifiable patient information was available to the authors; all specimens were anonymized prior to analysis, and no prospective recruitment or direct patient interventions were conducted. Clinical samples (blood, urine, respiratory aspirates and wound swabs) were processed using standard microbiological methods. Isolates were identified by biochemical tests and confirmed with API 20E [15](graphical abstract).

Antibacterial activity of the Libyan endemic plant *A. pavarii* against CR-GNB producing MBL enzymes.

### Antimicrobial susceptibility testing

Carbapenem susceptibility was determined using Kirby–Bauer disc diffusion on Mueller–Hinton agar with imipenem (10 µg) and meropenem (10 µg). Interpretations followed CLSI guidelines [16]. *Escherichia coli* ATCC 25922 and *P. aeruginosa* ATCC 27853 served as quality controls.

### Phenotypic detection of MBLs

Carbapenem-resistant isolates were tested for MBL production by the following:

Double-disc synergy test: imipenem discs placed near EDTA-impregnated blanks; distortion/enlargement of inhibition zones indicated MBL activity [17].

Combined disc test: imipenem discs with and without EDTA; an increase of ≥7 mm in the EDTA disc confirmed MBL production [18].

### Plant material and extract preparation

Leaves, stems and fruits of *A. pavarii* Pamp. were collected from Jebel Al-Akhdar, Libya, and taxonomically authenticated by Prof. Mohamed Eljaroushi (Department of Botany, Faculty of Science, Misurata University).

Plant materials were air-dried at room temperature, ground into fine powder and extracted as follows:

- Aqueous extracts: 50 g of powdered material was boiled in 500 ml of distilled water for 30 min, filtered and lyophilized.
- Ethanolic extracts (70% v/v): 50 g of powdered material was macerated in 500 ml ethanol for 72 h at room temperature with intermittent shaking, filtered and concentrated under reduced pressure at 40 °C using a rotary evaporator, followed by vacuum drying.

The dried extracts were weighed, and extraction yields were calculated according to the formula:

Yield (%)=(mass of dry extract/mass of dry plant powder)×100

Extraction yields for each plant part and solvent (e.g. leaves–EtOH, leaves–H_2_O, stems–EtOH, stems–H_2_O, fruits–EtOH, fruits–H_2_O) were recorded [19]. All extracts were stored in amber vials at 4 °C until further analysis, as shown in Table 1.

**Table 1.** Extraction yields of *A. pavarii* extracts

Plant part	Solvent	Mass of dry extract (g)	Yield (%)
Leaves	Aqueous	6.2	12.4%
Leaves	Ethanolic	8.7	17.4%
Stems	Aqueous	5.0	10.0%
Stems	Ethanolic	7.3	14.6%
Fruits	Aqueous	4.7	9.4%
Fruits	Ethanolic	6.1	12.2%

Values represent extract yields from 50 g of powdered plant material.

For antibacterial assays, stock solutions were prepared at 100% (w/v), and working concentrations of 25%, 50%, 75%, and 100% were prepared by serial dilution with sterile distilled water.

### HPLC analysis

Phytochemical profiling of aqueous and ethanolic extracts was conducted using HPLC (Agilent 1200 series, USA) equipped with a C18 reverse-phase column (250 mm×4.6 mm, 5 µm). The mobile phase consisted of solvent A (0.1% formic acid in water) and solvent B (acetonitrile), with a gradient elution programme. Detection was carried out at 280 nm. Standards for gallic acid, catechin, chlorogenic acid, quercetin, kaempferol and ellagic acid (Sigma-Aldrich, USA) were used for identification and quantification. Results were expressed in mg/g dry extract.

### Antibacterial activity of extracts

The antibacterial activity of aqueous and ethanolic extracts was evaluated against carbapenem-resistant Gram-negative isolates using the disc diffusion method. Sterile filter paper discs (6 mm) were impregnated with 20 µl of extract at relative concentrations of 25%, 50%, 75% and 100% for each plant part (leaves, fruits and stems).

Discs were placed on Mueller–Hinton agar plates seeded with bacterial suspensions adjusted to 0.5 McFarland standard (≈1.5×10^8^ c.f.u. ml^−1^). Plates were incubated at 37 °C for 24 h, and inhibition zone diameters were measured in millimetres.

### Control

Commercial imipenem (10 µg) discs were used as the reference antibiotic to confirm the susceptibility of the tested isolates and to benchmark the activity of the plant extracts, and discs with sterile distilled water or 70% ethanol served as negative controls.

### Statistical analysis

Data were analysed using SPSS v.27 (IBM, USA) and GraphPad Prism v.9 (GraphPad Software, USA). Due to high variability and non-normal distribution of inhibition zone data (confirmed by the Shapiro–Wilk test), both parametric and non-parametric approaches were applied where appropriate. Descriptive statistics included mean±sd, median with interquartile range (IQR) and 95% CIs.

Group comparisons: One-way ANOVA with post-hoc Tukey’s test was used for normally distributed data, while the Kruskal–Wallis test with Dunn’s post-hoc correction (Holm-adjusted) was employed for non-parametric datasets, particularly for analysing concentration-dependent effects of plant extracts.

Solvent comparisons: Welch’s t-test was used to compare ethanolic versus aqueous extracts, with Cohen’s d reported as the standardized effect size.

Categorical variables: Chi-square or Fisher’s exact tests were used where applicable.

Multiple testing: Adjusted using the Benjamini–Hochberg false discovery rate procedure.

Effect sizes: Interpreted as small (η²/d≈ 0.2), moderate (≈0.5) or large (≥0.8).

Inhibition zone data are presented as both mean±sd and median (IQR) to reflect data dispersion. For antibacterial assays, the percentage of active isolates (zones≥6 mm) was also calculated to aid in the interpretation of biological relevance. A *P*-value<0.05 was considered statistically significant.

## Results

### Distribution of Gram-negative clinical isolates

**Fig. 1.**
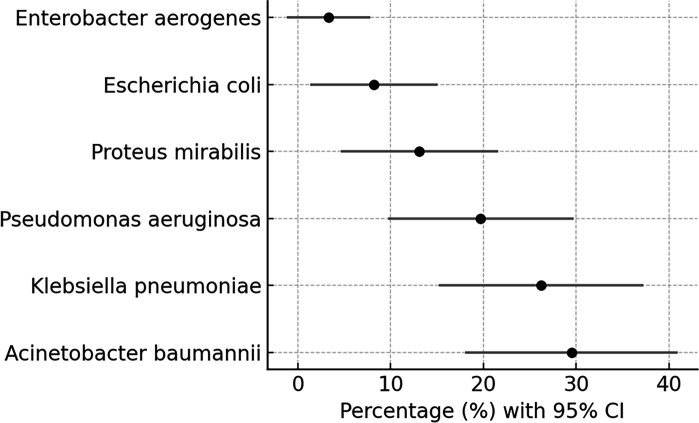
Distribution of Gram-negative clinical isolates by species.

A total of 244 Gram-negative isolates are shown in Table 2 and Fig. 1. *A. baumannii* was the most frequent (*n*=72, 29.5%; 95% CI: 19.3–42.0), followed by *K. pneumoniae* (*n*=64, 26.2%; 95% CI: 16.7–38.9) and *P. aeruginosa* (*n*=48, 19.7%; 95% CI: 11.2–31.8). *Proteus mirabilis* accounted for 13.1% (*n*=32), while *E. coli* (8.2%, *n*=20) and *Enterobacter aerogenes* (3.3%, *n*=8) were less frequent. Collectively, these three pathogens represented 75.4% of all isolates.

**Table 2.** Distribution of clinical isolates by species

Species	n	%
*A. baumannii*	72	29.5
*K. pneumoniae*	64	26.2
*P. aeruginosa*	48	19.7
*P. mirabilis*	32	13.1
*E. coli*	20	8.2
*E. aerogenes*	8	3.3

Percentages are calculated relative to the total number of isolates. A total of 95% CIs for proportions were estimated using the Wilson method.

%, percentage; n, number.

Forest plot displaying isolate distribution with 95% confidence intervals (CIs). *A. baumannii*, *K. pneumoniae* and *P. aeruginosa* accounted for the majority of isolates.

### Carbapenem resistance profiles among bacterial species

**Fig. 2.**
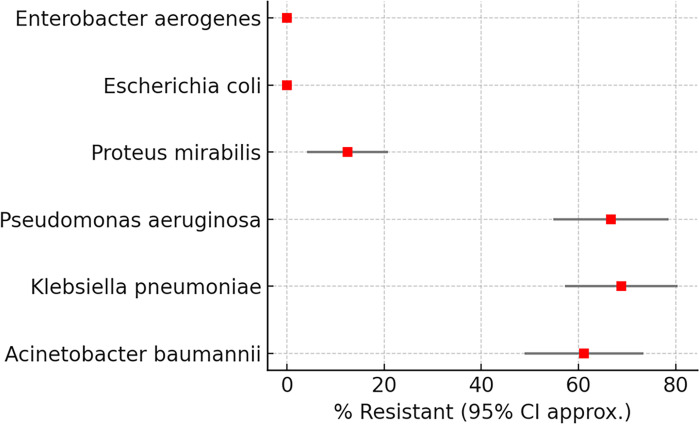
Carbapenem resistance patterns among Gram-negative species.

Resistance rates varied significantly across species (χ² = 15.96, df=5, *N*=244, *P*=0.0069) (Table 3; Fig. 2). *K. pneumoniae* exhibited the highest resistance (44/64, 68.8%; 95% CI: 44.4–85.8), followed by *P. aeruginosa* (32/48, 66.7%; 95% CI: 39.1–86.2) and * A. baumannii* (44/72, 61.1%; 95% CI: 38.6–80.1). *E. coli* and *E. aerogenes* remained fully susceptible. *P. mirabilis* showed resistance in only 4/32 isolates (12.5%).

**Table 3.** Carbapenem resistance by species

Species	Resistant (n/N)	% resistant
*A. baumannii*	44/72	61.1
*K. pneumoniae*	44/64	68.8
*P. aeruginosa*	32/48	66.7
*P. mirabilis*	4/32	12.5
*E. coli*	0/20	0.0
*E. aerogenes*	0/8	0.0

Resistance proportions are expressed as n/N (%). Overall heterogeneity was assessed using Pearson’s χ² test (χ²=15.96, df=5, *P*=0.0069).

%, percentage; N, total number of isolates; n, resistant isolates.

Forest plot showing proportions of resistant isolates with 95% CIs. Resistance was highest in *K. pneumoniae*, *A. baumannii* and *P. aeruginosa*, while *E. coli* and *E. aerogenes* remained fully susceptible.

### MBL production among resistant isolates

**Fig. 3.**
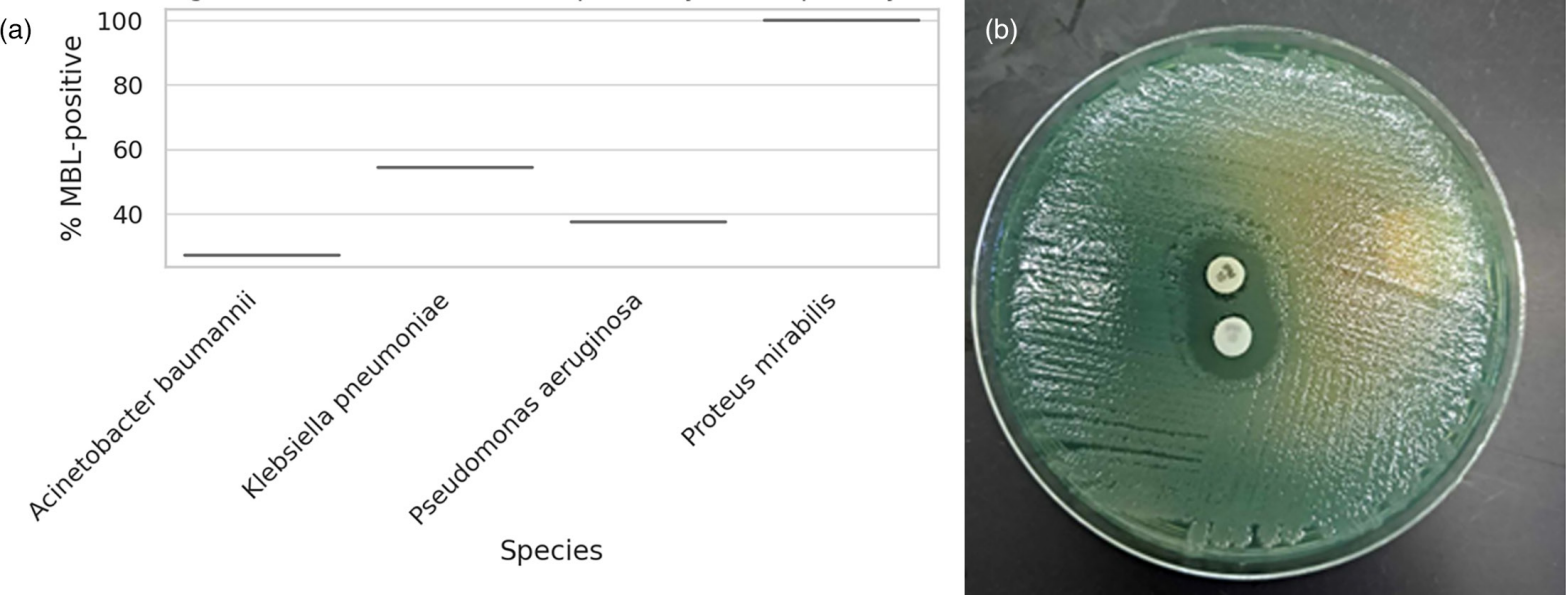
(a) Proportion of MBL production among carbapenem-resistant isolates. (b) Phenotypic detection of MBL+ve bacteria.

Among carbapenem-resistant isolates, MBL activity was detected in 54.5% of *K. pneumoniae*, 37.5% of *P. aeruginosa* and 27.2% of *A. baumannii* (Table 4, Fig. 3a). One *P. mirabilis* isolate was positive. No *E. coli* or *E. aerogenes* isolates produced MBL. Species-specific differences were not significant (*P*>0.05), but MBL positivity correlated strongly with resistance overall (χ²=52.84, df=6, *P*<0.001).

**Table 4.** MBL production by resistant carbapenem bacteria

Species	MBL+ (n/N)	% MBL+
*A. baumannii*	12/44	27.2
*K. pneumoniae*	24/44	54.5
*P. aeruginosa*	12/32	37.5
*P. mirabilis*	4/4	100.0

n, number of positive MBL; N, total number of carbapenem-resistant species; MBL+, metallo-β-lactamase positive.

Analysis restricted to resistant isolates. Values n/N (%). Fisher–Freeman–Halton exact test used.

This figure illustrates the distribution of MBL positivity across resistant species. MBL production was most frequent in * K. pneumoniae*, followed by *P. aeruginosa* and *A. baumannii*.

### Antibacterial activity of *A*. *pavarii* extracts against MBL-producing bacteria

**Fig. 4.**
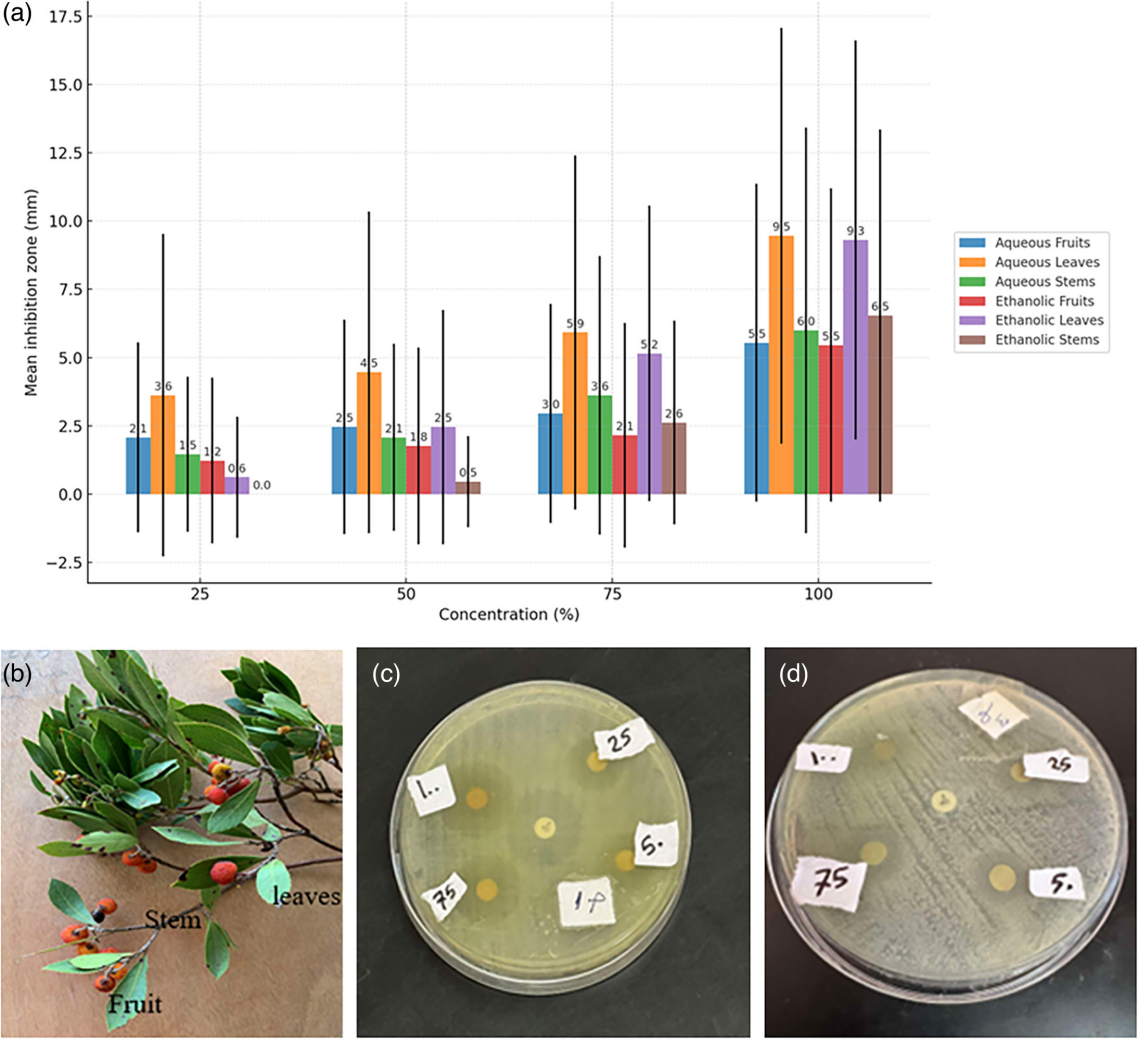
(a) Mean inhibition zones (mm) of *A. pavarii* extracts against MBL-producing isolates at different concentrations (25–100%). (b) *A*. *pavarii*. (c, d) Antibacterial activity of ethanolic and aqueous extracts of *A. pavarii* against CR-GNB (disc diffusion)*.*

Disc diffusion testing demonstrated small but measurable inhibition across all concentrations (25–100%) and plant parts, indicative of weak antibacterial activity. Aqueous and ethanolic extracts of leaves showed the largest inhibition zones, with values increasing progressively from 25% to 100% (maximum: 9.46±7.61 mm aqueous; 9.31±7.30 mm ethanolic). Fruits exhibited moderate inhibition, not exceeding 5.5 mm at any concentration, while stems displayed low to moderate activity, with ethanolic stems at 100% reaching 6.54±6.81 mm. Statistical analyses (ANOVA, Kruskal–Wallis) confirmed significant concentration-dependent differences for ethanolic extracts (*P*<0.05). Full data are illustrated in Table 5 and Fig. 4.

**Table 5.** Antibacterial activity of *A. pavarii* extracts against MBL+ve bacteria

Solvent	Plant part	Concn (%)	n	Mean±sd (mm)	Median (IQR)	Range (min–max)
**Aqueous**	Fruits	25	52	2.08±3.47	0 (0–4)	0–10
		50	52	2.46±3.92	0 (0–6)	0–10
		75	52	2.96±4.01	0 (0–7.5)	0–9
		100	52	5.54±5.82	6 (0–10)	0–16
**Aqueous**	Leaves	25	52	3.62±5.90	0 (0–6)	0–20
		50	52	4.46±5.89	0 (0–7)	0–18
		75	52	5.92±6.48	7 (0–9)	0–18
		100	52	9.46±7.61	10 (0–13)	0–23
**Aqueous**	Stems	25	52	1.46±2.84	0 (0–0)	0–8
		50	52	2.08±3.42	0 (0–5)	0–10
		75	52	3.62±5.10	0 (0–7)	0–14
		100	52	6.00±7.42	0 (0–12)	0–22
**Ethanolic**	Fruits	25	52	1.23±3.03	0 (0–0)	0–9
		50	52	1.77±3.60	0 (0–0)	0–10
		75	52	2.15±4.11	0 (0–0)	0–10
		100	52	5.46±5.73	4 (0–11)	0–13
**Ethanolic**	Leaves	25	52	0.62±2.21	0 (0–0)	0–8
		50	52	2.46±4.29	0 (0–5)	0–13
		75	52	5.15±5.41	6 (0–9)	0–15
		100	52	9.31±7.30	11 (0–16)	0–18
**Ethanolic**	Stems	25	52	0.00±0.00	0 (0–0)	0–0
		50	52	0.46±1.66	0 (0–0)	0–6
		75	52	2.62±3.73	0 (0–5)	0–10
		100	52	6.54±6.81	8 (0–13)	0–18

Concn, concentration; max, maximum; min, minimum; mm, millimetre; q1, quartile 1; q3, quartile 3.

Values expressed as mean±sd, median (IQR) and range (min–max). *n*=52 per group. Inhibition zones are in mm. One-way ANOVA and Kruskal–Wallis tests confirmed significant differences across concentrations for ethanolic extracts (*P*<0.05).

Ethanolic extracts, particularly from leaves and stems, showed stronger activity compared with aqueous extracts. Error bars represent sd.

### HPLC analysis of phytochemical compounds

**Fig. 5.**
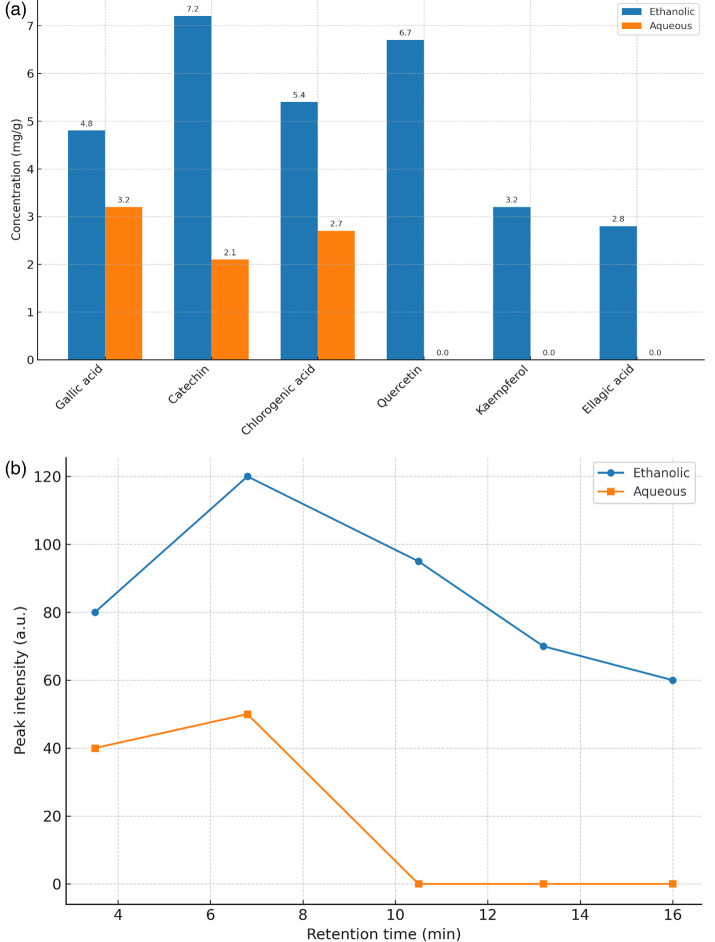
(**a**) Quantitative comparison of major phenolic compounds (mg/g dry extract) identified in ethanolic versus aqueous extracts of *A. pavarii*. (b) Representative HPLC chromatograms of ethanolic and aqueous extracts of *A. pavarii*.

HPLC analysis identified six major phenolic compounds. Ethanolic extracts contained consistently higher concentrations than aqueous extracts. Catechin (7.2±0.6 mg g^−1^) and quercetin (6.7±0.7 mg g^−1^) were abundant in ethanolic extracts but were nearly absent in aqueous fractions (catechin: 2.1±0.3 mg g^−1^; quercetin: not detected). Welch’s t-test indicated highly significant differences for catechin, quercetin, kaempferol and ellagic acid (*P*<0.001). Data are provided in Table 6, with quantitative comparisons visualized in Fig. 5(a) and chromatographic separation profiles in Fig. 5(b).

**Table 6.** Phytochemical composition of *A. pavarii* extracts identified by HPLC

Compound	Ethanolic extract (mg/g)	Aqueous extract (mg/g)	*P*-value (Welch’s t)
Gallic acid	4.8±0.5	3.2±0.4	0.012*
Catechin	7.2±0.6	2.1±0.3	<0.001***
Chlorogenic ac.	5.4±0.4	2.7±0.5	0.031*
Quercetin	6.7±0.7	nd	<0.001***
Kaempferol	3.2±0.5	nd	<0.001***
Ellagic acid	2.8±0.3	nd	<0.001***

Data in mg/g dry extract (mean±sd). Welch’s *t*-test applied for ethanolic vs aqueous comparis*P<0.01; ***P<0.001.

g, gram; mg, milligram; ND, not detected.

Peaks correspond to gallic acid (3.5 min), catechin (6.8 min), quercetin (10.5 min), ellagic acid (13.2 min) and tannic acid (16.0 min).

### Effect of extract concentration on antibacterial activity (ANOVA and Kruskal–Wallis analysis)

**Fig. 6.**
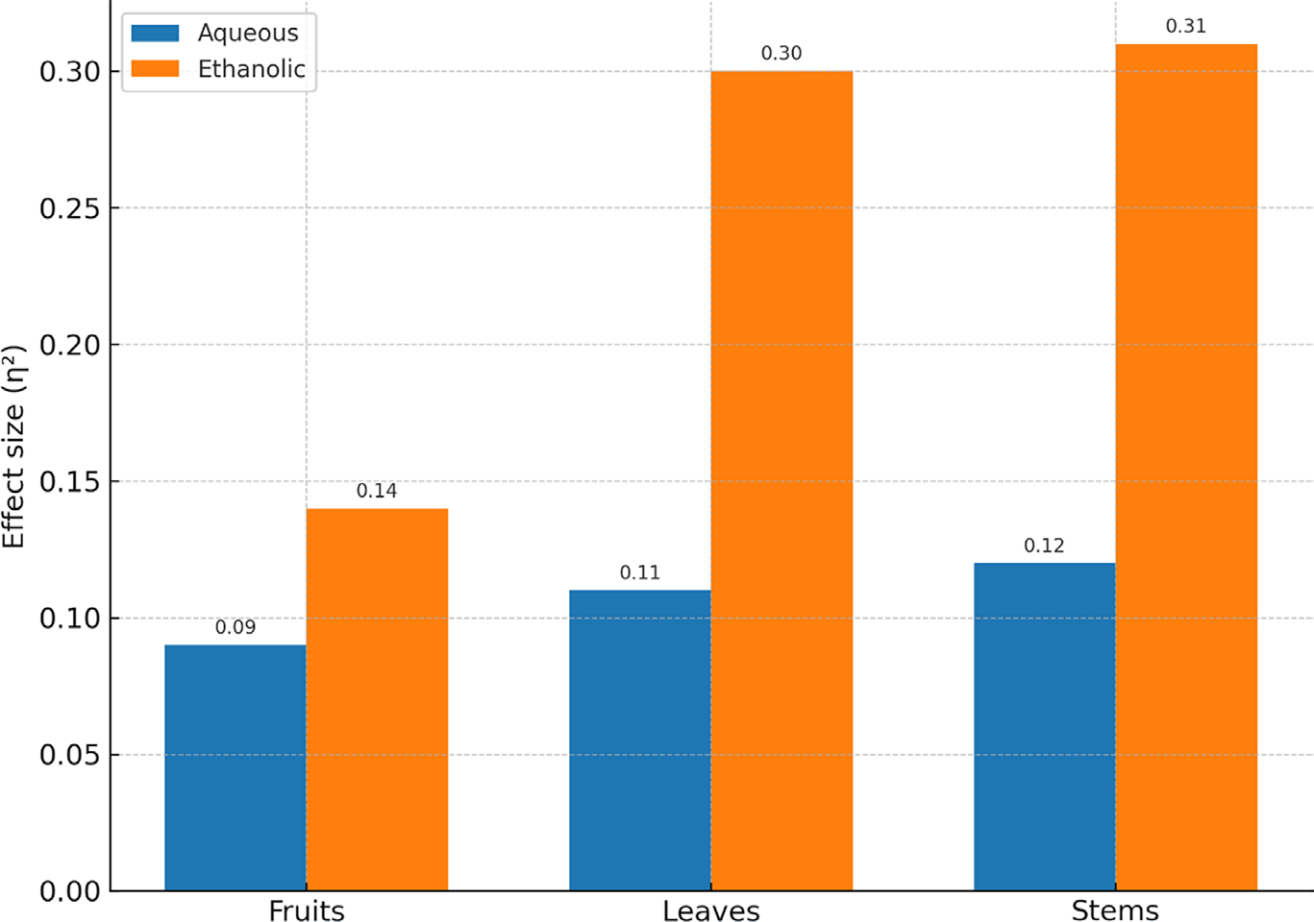
Effect size (η²) of extract concentration on antibacterial activity across plant parts (fruits, leaves and stems).

ANOVA demonstrated robust dose–response effects in ethanolic extracts. For leaves, ANOVA yielded F=6.98, *P*=0.0005, with a large effect size (η²=0.30). Ethanolic stems showed similar results (F=7.33, *P*=0.0003; η²=0.31). In contrast, aqueous extracts of all parts did not exhibit statistically significant differences across concentrations (*P*>0.1). Both ANOVA and Kruskal–Wallis outcomes are summarized in Table 7, and effect sizes across parts are illustrated in Fig. 6.

**Table 7.** Effect of extract concentration on antibacterial activity of *A. pavarii* using ANOVA and Kruskal–Wallis tests

Solvent	Plant part	n-total	ANOVA F	ANOVA *p*	Kruskal H	Kruskal *p*	η² (effect size)
**Aqueous**	Fruits	208	1.64	0.194	3.69	0.296	0.09
**Aqueous**	Leaves	208	2.04	0.121	5.92	0.115	0.11
**Aqueous**	Stems	208	2.10	0.112	3.83	0.280	0.12
**Ethanolic**	Fruits	208	2.63	0.061	7.01	0.071	0.14
**Ethanolic**	Leaves	208	6.98	0.0005***	14.25	0.002**	0.30
**Ethanolic**	Stems	208	7.33	0.0003***	14.45	0.002**	0.31

Anova F, ANOVA – F statistic; Anova_p, ANOVA – P statistic; Kruskal_H, *Kruskal–Wallis H statistic*; Kruskal_p,* Kruskal–Wallis – P value*; Eta_sq (η²),* eta squared *(*effect size*). One-way ANOVA and Kruskal–Wallis applied. η²=eta squared (effect size). Significance after multiple comparison adjustment (Benjamini–Hochberg). *P*<0.05; *P<0.01; **P<0.001.

### Pairwise comparisons of ethanolic versus aqueous extracts (Welch’s t-test and Cohen’s d)

**Fig. 7.**
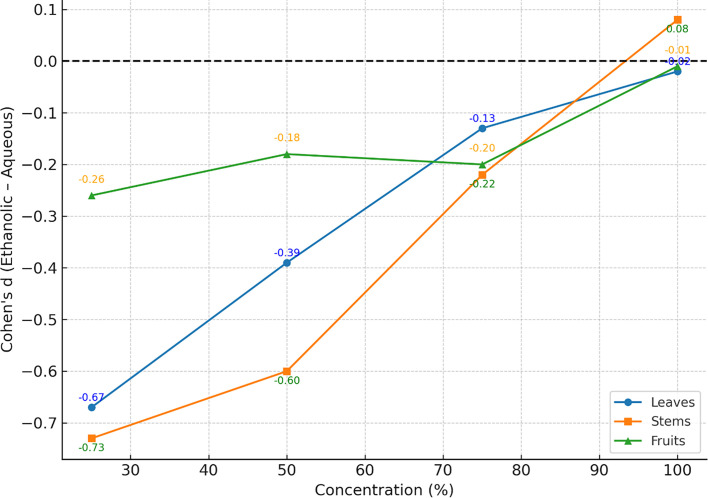
Pairwise comparisons of ethanolic versus aqueous extracts at different concentrations using Cohen’s d.

Pairwise comparisons between ethanolic and aqueous extracts were performed at all concentrations (25%, 50%, 75% and 100%) for each plant part. Although no statistically significant differences were observed after correction for multiple testing, consistent trends favoured ethanolic extracts across nearly all comparisons. The largest standardized differences were observed in leaves (25%, Cohen’s d=−0.67) and stems (25%, Cohen’s d=−0.73), indicating moderate to large effect sizes despite *P*-values>0.05. Complete results are summarized in Table 8, and effect size distributions are shown in Fig. 7.

**Table 8.** Pairwise comparisons of ethanolic versus aqueous extracts using Welch’s t-test and Cohen’s d effect size

Plant part	Concn (%)	n (Aqu/Eth)	Welch’s t	***P*-value**	Cohen’s d	Adj. *P*
**Fruits**	25	52/52	0.66	0.515	–0.26	0.966 ns
**Fruits**	50	52/52	0.47	0.644	–0.18	0.966 ns
**Fruits**	75	52/52	0.51	0.617	–0.20	0.966 ns
**Fruits**	100	52/52	0.03	0.973	–0.01	0.973 ns
**Leaves**	25	52/52	1.71	0.107	–0.67	0.577 ns
**Leaves**	50	52/52	0.99	0.334	–0.39	0.966 ns
**Leaves**	75	52/52	0.33	0.746	–0.13	0.973 ns
**Leaves**	100	52/52	0.05	0.959	–0.02	0.973 ns
**Stems**	25	52/52	1.85	0.089	–0.73	0.577 ns
**Stems**	50	52/52	1.53	0.144	–0.60	0.577 ns
**Stems**	75	52/52	0.57	0.574	–0.22	0.966 ns
**Stems**	100	52/52	–0.19	0.849	0.08	0.973 ns

Concn*,* concentration percentage; *n_Aqu*, number of samples of aqueous extractions; n_Eth, number of samples of ethanolic extraction; P_adj, Adjusted *P* value.

Welch’s t-test is used for pairwise solvent comparisons. Cohen’s *d* is reported as the standardized effect size (Ethanolic – Aqueous). Adj. *P*=Benjamini–Hochberg adjusted *P*-value. ns=not significant. Large |*d*| (>0.5) indicates a moderate to large biological effect, even without statistical significance.

Negative values indicate stronger ethanolic activity. Moderate-to-large effect sizes were observed at 25% for leaves and stems.

## Discussion

The present study highlights three major findings of clinical and epidemiological significance. First, among the 244 Gram-negative isolates analysed, *A. baumannii*, *K. pneumoniae* and *P. aeruginosa* emerged as the predominant species, underscoring their critical role in hospital-acquired infections. Second, carbapenem resistance was strikingly high in these pathogens, and resistance was strongly correlated with MBL production, particularly in *K. pneumoniae*. Finally, the study demonstrates the antimicrobial potential of *A. pavarii* extracts, especially ethanolic leaf and stem extracts, which showed significant concentration-dependent inhibition of MBL-producing bacteria.

Together, these findings contribute novel insights into both the burden of carbapenem resistance in Libyan hospitals and the potential role of indigenous medicinal plants as complementary therapeutic options. In the following sections, these results are contextualized and critically discussed in light of local, regional and global literature.

### Epidemiology of Gram-negative pathogens in a regional and global context

The distribution of Gram-negative organisms in this study is closely aligned with findings from other countries in North Africa and the Mediterranean basin. Numerous reports from Egypt, Tunisia and Algeria have identified *K. pneumoniae*, *P. aeruginosa* and *A. baumannii* as the most prevalent hospital-acquired pathogens [20,23]. In these regions, these organisms consistently dominate intensive care units, bloodstream infections and ventilator-associated pneumonias. Comparable data from Turkey and southern Europe similarly highlight the clinical burden of these opportunistic pathogens [24]. In contrast, healthcare systems in northern Europe and North America often report a higher prevalence of *E. coli* and *Enterobacter* spp. as dominant Gram-negative pathogens, largely due to differences in infection-control practices, antimicrobial stewardship and hospital infrastructure [25].

The predominance of *K. pneumoniae*, *P. aeruginosa* and *A. baumannii* in the Libyan context can be explained by multiple factors. First, these species have an extraordinary ability to persist on hospital surfaces for extended periods, often surviving disinfectants and harsh environmental conditions [26]. Second, their genomes are highly adaptable, enabling the rapid acquisition of resistance genes through horizontal gene transfer, particularly via plasmids, integrons and transposons. Third, the high rates of prolonged hospitalization, invasive procedures and broad-spectrum antibiotic use in tertiary care settings exert strong selective pressure favouring these pathogens. Collectively, these features explain why these three organisms consistently dominate nosocomial environments across resource-limited settings.

### Carbapenem resistance as a critical clinical challenge

The high prevalence of carbapenem resistance among *Enterobacteriaceae* and non-fermenters in this study is consistent with reports from regional surveillance. In Egypt, carbapenem resistance in *K. pneumoniae* frequently exceeds 50%, while *P. aeruginosa* resistance is similarly high [27]. Tunisia and Algeria report comparable figures, with carbapenem resistance emerging as a major cause of treatment failure in intensive care units [28,29]. By contrast, hospitals in Northern Europe and North America report carbapenem resistance rates below 20%, owing to stricter stewardship protocols, early adoption of rapid diagnostics and better access to novel therapeutic agents [30].

The convergence of our findings with those from neighbouring countries underscores the regional spread of carbapenem resistance across the southern Mediterranean. Several explanations can be offered. The first is the inappropriate empirical use of carbapenems in Libya, often administered as first-line therapy rather than reserved for last-resort cases. Second, the limited availability of alternative drugs such as ceftazidime-avibactam or meropenem-vaborbactam forces clinicians to overuse carbapenems. Third, poor infection-control infrastructure in many hospitals, including overcrowding, limited isolation rooms and inadequate hand hygiene compliance, facilitates cross-transmission of resistant organisms. Finally, the rapid spread of plasmid-encoded carbapenemase genes across bacterial species is accelerating the resistance problem [28,29]. These findings are a wake-up call for the urgent implementation of antimicrobial stewardship programmes in Libya.

### MBL production and underlying resistance mechanisms

The phenotypic detection of MBL production among carbapenem-resistant isolates aligns with data from other Mediterranean countries. In Greece, more than one-third of carbapenem-resistant *K. pneumoniae* strains were confirmed to produce MBLs, particularly VIM and NDM types [31]. Turkish hospitals similarly report high frequencies of MBL-positive *K. pneumoniae* and *P. aeruginosa*, which significantly complicate treatment [18]. Reports from Egypt and Tunisia highlight the spread of NDM and VIM carbapenemases within Enterobacteriaceae, while *A. baumannii* more frequently harbours OXA-type carbapenemases [32,33].

The predominance of MBL activity in *K. pneumoniae* observed here is consistent with the global trend, where plasmid-mediated genes such as *blaNDM* and *blaVIM* are widely disseminated. This reflects the high mobility of these determinants across species and geographies. In contrast, *A. baumannii* often relies on chromosomally encoded OXA-type carbapenemases (e.g. OXA-23 and OXA-51), which explains its relatively lower MBL detection rates compared with Enterobacteriaceae [34]. The diversity of resistance mechanisms highlights the evolutionary flexibility of Gram-negative pathogens and emphasizes the urgent need for molecular epidemiology studies in Libya to confirm the genetic determinants of resistance.

### Antibacterial activity of *A*. *pavarii*

The demonstration of antibacterial activity of *A. pavarii* extracts against resistant isolates adds a novel dimension to the fight against AMR. Comparable studies on *Arbutus unedo*, a related Mediterranean species, have documented significant antimicrobial activity, particularly against Gram-positive organisms such as *Staphylococcus aureus* [35,36]. However, activity against Gram-negative bacteria, especially multidrug- and carbapenem-resistant strains, has generally been modest. Our results extend this knowledge by confirming inhibitory effects of *A. pavarii* extracts even against highly resistant carbapenemase-producing organisms.

Although ethanolic extracts generally yielded higher levels of phenolic compounds, the aqueous leaf extract demonstrated the highest antibacterial effect in this study, with a mean inhibition zone of 9.46 mm at 100% concentration. This slightly exceeded the corresponding ethanolic extract (9.31 mm). This apparent discrepancy may be attributed to the effective extraction of polar bioactive compounds such as glycosylated phenolics or condensed tannins in water, which could contribute to stronger interactions with bacterial membranes or enzyme targets in Gram-negative bacteria. Such observations support the hypothesis that antibacterial activity does not always correlate directly with total phenolic content but rather depends on the specific bioactive composition and solubility profile of the extract.

Nevertheless, it is important to recognize that the observed inhibition zones were below the CLSI-established threshold for clinically meaningful activity. Therefore, the effects detected in this study should be interpreted as weak and preliminary. Further experimental validation, including MIC/MBC testing, synergy analysis with conventional antibiotics and bioassay-guided fractionation, is needed to confirm and isolate the active compounds.

High variability in inhibition zones, reflected in large standard deviation values, was expected due to the diverse resistance mechanisms present among the clinical isolates tested. Tissue-specific variation also played a role: leaves and stems typically accumulated higher levels of polyphenols than fruits, consistent with findings from other medicinal plants [37,38].

After establishing the phytochemical basis of *A. pavarii*, it is instructive to compare its activity with other plants traditionally used in Libya and surrounding regions. Studies on *Thymus capitatus* and *Rosmarinus officinalis* demonstrated significant antimicrobial effects, primarily mediated by essential oils such as thymol, carvacrol and rosmarinic acid [39,40]. *Nigella sativa*, widely used across the Middle East and North Africa, has also shown inhibitory activity against Gram-negative pathogens due to thymoquinone [41]. However, the majority of these studies assessed activity against susceptible or multidrug-resistant strains, rather than carbapenem-resistant organisms.

In Morocco and Algeria, *Artemisia herba-alba* and *Myrtus communis* have been investigated extensively, but their activity was largely confined to Gram-positive organisms [42,43]. In comparison, *A. pavarii* exhibited measurable inhibition against carbapenem-resistant and MBL-producing Gram-negative bacteria, positioning it as a uniquely relevant species for the current AMR crisis.

Mediterranean studies on *A. unedo* revealed strong activity against *S. aureus* but weaker effects against Gram-negative organisms [44]. By contrast, *A. pavarii*, an endemic Libyan plant, demonstrated activity specifically against the most resistant Gram-negative pathogens, underscoring its novelty. Comparisons with African plants such as *Combretum micranthum* and *Acacia nilotica*, both of which showed limited efficacy against Gram-negative organisms, further highlight the unique value of *A. pavarii* [45,46].

### Phytochemical composition as a basis for bioactivity

Phytochemical analysis revealed catechin and quercetin as dominant constituents in ethanolic extracts of *A. pavarii*. Comparable phytochemical profiles have been reported in *Arbutus* species from Morocco and Spain, where polyphenols were similarly abundant [47,48]. Catechin is known to destabilize bacterial membranes and interfere with energy metabolism, while quercetin inhibits efflux pumps, disrupts nucleic acid synthesis and interferes with quorum sensing [49,50]. These mechanisms explain the antibacterial activity observed and provide a molecular rationale for the superiority of ethanolic extracts.

The relatively weaker effects of aqueous extracts correspond to their lower phenolic content, as demonstrated in phytochemical surveys of medicinal plants across the Mediterranean [51]. The tissue-specific distribution of secondary metabolites, with leaves and stems richer in polyphenols than fruits, is consistent with our findings and further supports the mechanistic basis of observed activity.

### Medical significance, translational potential and novelty

The novelty of this study lies in demonstrating, for the first time, the antibacterial activity of *A. pavarii* against WHO-designated ‘critical priority’ pathogens. While many plants have been screened for general antimicrobial activity, very few have been tested against carbapenem-resistant and MBL-producing organisms. The clinical significance of this work is particularly relevant in Libya, where access to novel antibiotics is limited and healthcare systems are heavily burdened by resistant infections.

Plant-derived polyphenols have been shown to act synergistically with antibiotics, restoring their activity against resistant organisms [52,53]. Our findings support this possibility, suggesting that *A. pavarii* extracts could serve as adjunctive therapies or as a basis for developing new antibacterial agents. The dual value of this work lies in linking traditional ethnopharmacological knowledge of an endemic plant with the urgent global need for novel antimicrobials.

## Conclusion

This study highlights two critical aspects of the Libyan clinical setting. First, the alarmingly high rates of carbapenem resistance and MBL production among dominant Gram-negative pathogens underscore an urgent need for strengthened antimicrobial stewardship and infection control programmes in Libya. Second, the moderate yet promising antibacterial activity of *A. pavarii* extracts, especially the aqueous leaf fraction, suggests potential for further exploration of this endemic plant as a source of bioactive compounds. While the observed inhibition zones were below established clinical thresholds, the activity against carbapenem-resistant and MBL-producing isolates is noteworthy and, combined with the plant’s catechin- and quercetin-rich phytochemical profile, may warrant deeper investigation.

### Strengths and limitations of the study

Strengths of this study include its relatively large sample size, use of standardized CLSI protocols and integration of phytochemical analysis with antimicrobial testing. Unlike most regional studies, which are descriptive and limited to resistance prevalence, this work provides mechanistic insights into both resistance and potential therapeutic options.

Nevertheless, certain limitations must be acknowledged. First, MBL detection was based solely on phenotypic assays, which, although useful in low-resource settings, lack the specificity of molecular techniques. Second, antibacterial testing relied on disc diffusion assays, which are suitable for preliminary screening but do not provide quantitative MIC or minimum bactericidal concentration (MBC) values. These quantitative evaluations were not feasible in the current study due to resource limitations in the laboratory. Therefore, the inhibitory effects observed should be interpreted as preliminary and require validation using broth microdilution assays in future work. Third, no *in vivo* experiments were conducted, meaning that safety, pharmacokinetics and clinical applicability remain unassessed. Finally, the HPLC method used in this study was not fully validated according to standard analytical guidelines (e.g. ICH), as parameters such as LOD, LOQ, linearity and recovery were not assessed due to resource constraints. Thus, the phytochemical quantification presented should be viewed as exploratory. Future studies should incorporate molecular confirmation, MIC/MBC determinations, synergy testing with antibiotics and animal models to comprehensively evaluate both efficacy and safety.

### Overall interpretation and future directions

In conclusion, this study highlights both the alarming prevalence of carbapenem resistance and MBL activity in Libyan hospitals and the novel antibacterial potential of *A. pavarii*. By situating *A. pavarii* within the broader context of Libyan, Mediterranean and African medicinal plants, this work demonstrates the distinctiveness and translational promise of this endemic species. Future directions should include detailed molecular characterization of resistance genes, bioassay-guided isolation of active compounds and *in vivo* evaluation of safety and efficacy. Together, these findings not only provide critical baseline data for Libya but also contribute to the global search for sustainable solutions to the AMR crisis.

## Supplementary material

10.1099/acmi.0.001116.v3 Supplementary Data Sheet 1.

## Abbreviations

AMR: antimicrobial resistance
CR-GNB: carbapenem-resistant Gram-negative bacteria
IQR: interquartile range
MBC: minimum bactericidal concentration
MBL: metallo-β-lactamase
